# Epidemic Response amidst Insecurity: Addressing the Ebola Virus Epidemic in the Provinces of North Kivu and Ituri

**DOI:** 10.29245/2578-3009/2023/S3.1102

**Published:** 2023-05-12

**Authors:** Tieman Diarra, Joseph Okeibunor, Amadou Baïlo Diallo, Nkechi Onyeneho, Barry Rodrigue, Michel N’da Konan Yao, Zabulon Yoti, Mamoudou Harouna Djingarey, Ibrahima Socé Fall, Abdou Salam Gueye

**Affiliations:** 1 Independent Consultant, Mali; 2 World Health Organization, Switzerland; 3 University of Nigeria, Nsukka

**Keywords:** Epidemic, Response, Attacks, Threats, Disruptions, Response teams, Response perception, Negotiation, Securing

## Abstract

This paper examines the impact of insecurity on the management of the Ebola virus disease epidemic in the Democratic Republic of the Congo provinces of North Kivu and Ituri. In these provinces, insecurity has been one of the biggest obstacles in the response to the Ebola outbreak. When the epidemic began, these provinces were already insecure—creating unfavorable circumstances for implementing epidemic response activities. While the ninth epidemic in the Equateur province was brought under control in record time, the same was not true for the tenth epidemic in North Kivu and Ituri. Since the epidemic began, teams were organized to address all aspects of the response. These response teams conducted extensive fieldwork, including epidemiological surveillance, risk communication and community involvement, infection prevention and control, vaccination, dignified and safe burials, care at transit centers and Ebola treatment centers, and medical and psychosocial care for the recovered. They faced confrontational reactions from the communities, which jeopardized their security. The insecure state of the provinces led to the destruction and damage of infrastructure, including healthcare facilities, which affected the ability of rescue teams to access people needing care as well as the resources they needed to care for the ill. Worse yet, the insecurity took other forms, including threatening and kidnapping members of the response teams, lodging protests against the response activities in towns or health zones, committing violence against teams responsible for safe and dignified burials, instigating altercations between community members and members of the response team, and encouraging general resistance by the population. This level of insecurity interrupted or even halted response activities in some areas—sometimes for more than two weeks, decreasing the efficiency of the response teams, particularly in monitoring contacts due to the inability to access certain communities. Additionally, certain acts of protest, such as community members handling bodies as a demonstration of their opposition to safe and dignified burials, likely intensified disease spread. However, the involvement of community leaders, at least, made dialogue and negotiation possible between the response teams and community members, as such efforts led to communities contributing to the security of personnel involved in the fight against the Ebola epidemic in North Kivu and Ituri provinces.

## Introduction

The fight against the Ebola virus disease (EVD) epidemic in the Democratic Republic of the Congo (DRC) provinces of North Kivu and Ituri erupted in an environment inundated with insecurity^1^. Such insecurity has been a major obstacle to the work of the teams responsible for responding to this epidemic. Communication, social mobilization and community involvement activities, vaccination, infection prevention and control, epidemiological surveillance, and dignified and safe burials were often disrupted or even halted for days or weeks in some areas (i.e., health areas, health zones, or towns). This has made the *response* teams’ work not only more complicated but also more dangerous as team members have often been detained and targeted^2^.

Violence against team members and the destruction of response infrastructure, such as the hand washing facilities and the patient sorting system contributed to disruptions in the response. In some cases, the security incidents that affected the response teams have decreased the pace of activities, interrupted or halted activities, made certain communities inaccessible, and even resulted in the complete abandonment of certain facilities by the staff^3,4^.

Community resistance to the response teams also complicated the response effort, resulting in failed efforts in achieving desired targets^5^. On February 27, 2019, the Médecins Sans Frontières (MSF)-run Ebola treatment centers in Katwa and Butembo in the DRC were attacked. Then again, on March 14, 2019, the Ebola treatment center in Biena was attacked. There were also a series of attacks on burial teams, infection prevention and control teams, and vaccination teams.

Such attacks have a significant and immediate impact on lives as they increase the risk of disease spread. The exact reasons for the resistance and attacks on treatment centers and response teams remain unclear. In a meeting to sensitize religious leaders on EVD in Goma, the participants insisted that the Ebola outbreak was not real, alleging that it was merely a rumor created by the political class to subdue the people.

This article focuses on the part that insecurity has played in the response. It does not focus on the impact of insecurity on the epidemiological situation but rather indicates how the disruption of response activities contributed to the slowing down of these activities. Insecurity also contributed to prolonging the epidemic as it increased the factors of disease exposure, such as unmonitored contacts and the handling of bodies by the population hostile to safe and dignified burials. The article draws on data collected using both qualitative and quantitative methodologies. It begins by discussing insecurity as an element of disturbance of the response. It describes incidents that have affected the implementation of response activities; in this context, the duration of the response is examined. Next, the perception of the response by the agents involved in the context of insecurity is analyzed. Finally, the relevance of security management for the success of the response is addressed.

The response activities could only be carried out if the teams responsible for their implementation were safe. This security was made possible via the involvement of community leaders, local authorities, and elected representatives in the response. Other security efforts were provided by DRC defense and security forces, the United Nations (UN) security system in the country, and the security agencies involved in the response to the EVD epidemic in the DRC.

The involvement of various agents in providing security for the response teams enabled the implementation of activities in all the sub-teams in the response, as well as at the coordination office in Goma. Dialogue and negotiation helped transform a population that was often hostile and averse to the response. Although previously a main source of insecurity, the population has since contributed to securing the activities and teams engaged in the response.

## Study Design and Methods

### Study Design

This study was designed to explore and document experiences and lessons around the response to the 10^th^ EVD outbreak in the North Kivu and Ituri provinces of the DRC. The study adopted a cross-sectional design with mixed-method techniques of data collection. The design allowed multiple windows of data harvesting, while the mixed method added benefits of both quantitative and qualitative approaches, guaranteeing the integrity and robust analysis and conclusion that this type of evaluation deserved.

### Selection of Study Area and Population:

The study was carried out in North Kivu and Ituri provinces where the 10^th^ EVD outbreak occurred in the DRC.

**Ituri** is 1 of the 26 provinces of the DRC. Its capital is the city of Bunia. The Ituri Rainforest is in this area. Ituri is located northeast of the Ituri River and on the western side of Lake Albert. It is a high plateau (2000–5000 meters) region that has a large tropical forest as well as savannah landscapes. The district has rare fauna, including the okapi, the national animal of the Congo. As for flora, an important species is Mangongo, whose leaves are used by the Mbuti, a pygmy ethnic group, to build their homes. The Ituri population is composed primarily of Alur, Hema, Lendu, Ngiti, Bira, and Ndo-Okebo, with differing figures on which one of the groups constitutes the majority population in the province. The Mbuti reside primarily in the Ituri forest near the Okapi Wildlife Reserve, although some Mbuti have been forced into urban areas by deforestation, overhunting, and violence. The Kilo-Moto gold mines are partly located in Ituri. Early in the 21st century, petroleum reserves were found by Heritage Oil and Tullow Oil on the shores of Lake Albert.

### North Kivu

(French: *Nord-Kivu*) is a province bordering Lake Kivu in eastern DRC. Its capital is Goma. North Kivu borders the provinces of Ituri to the north, Tshopo to the northwest, Maniema to the southwest, and South Kivu to the south. To the east, it borders the countries of Uganda and Rwanda. The province consists of three cities—Goma, Butembo, and Beni—and six territories—Beni, Lubero, Masisi, Rutshuru, Nyiragongo, and Walikale. It is home to the Virunga National Park, a World Heritage Site containing the endangered mountain gorillas. Except for the heightened insecurity and isolation due to rebel activities, North Kivu shares similar demographics with Ituri. The province is politically unstable, and since 1998, it has been one of the flashpoints of the military conflicts in the region.

The **2018 or 10^th^ Kivu Ebola outbreak** began on August 1, 2018, when four patients were confirmed to have tested positive for the Ebola virus in the eastern region of Kivu in the DRC^6,7,8^. The Kivu outbreak included Ituri Province after the first case was confirmed on August 13, 2018^9^. This outbreak started just days after the end of the 2018 Équateur province DRC EVD outbreak^10,11^.

The affected province and general region are currently facing a military conflict, which is hindering treatment and prevention efforts. The World Health Organization’s (WHO) Deputy Director-General for Emergency Preparedness and Response has described the combination of military conflict and civilian distress as a potential “perfect storm” that could lead to a rapid worsening of the outbreak^12^. Due to the deteriorating situation in North Kivu and surrounding areas, the WHO raised the risk assessment at the national and regional level from “high” to “very high” on September 27, 2019^12^.

The study population comprised adults (defined as individuals 18 years old or older) living in the community as well as response team members. A 2010 estimate put the population of North Kivu at 5,767,945. With an annual growth rate of 3.2%, the province’s population in 2019 was estimated at 7,658,406 and 5,360,884 for the general and adult populations, respectively. A 2005 estimate put the population of Ituri at 4,037,561. For 2019, the populations were estimated as 6,275,305 and 4,392,714 for the general and adult populations, respectively.

The response team consisted of over 10,000 persons. These individuals were in different response pillars, namely, surveillance, risk communication, social anthropology, vaccination, infection prevention and control, treatment and care, safe and dignified burial, security, logistics, and administration among others.

## Sample size estimation and Sampling strategy

### Sample size

This was an exploratory study. However, to achieve statistical conclusions on certain indicators of perceptions and practices, juxtaposed with relevant demographic characteristics, a sample of the study population was taken. With an assumed 50% chance of having accepted Ebola control interventions at a confidence interval of 95% with an error margin of 5%, a sample size of 384 was computed for the quantitative study. Therefore, for the two provinces, this totaled 768, which was rounded up to 800 to make allowance for losses. The sample size for the qualitative study depended on the saturation of information after the first two interviews conducted were collected from each category of respondents.

### Sampling strategy

A multi-stage sampling technique was adopted in selecting the communities, households, and respondents in this study. Two administrative areas (epicenters of EVD outbreak within each province) were purposively selected. Ten communities were randomly selected from each of the two administrative areas in the province.

### Selection of the households and respondents

The center of the selected communities was the reference point from which the team determined the first route and first household. Thereafter, the team visited the next household to the right and then continued until the number of households to be sampled was reached. Where there was a *cul-de-sac*, the step was retraced, and a turn to the left and then to the right was made to continue the sampling process.

Once in a selected household, an adult (≥ 18 years) was randomly selected for inclusion as a participant in the study. The sex of the participants was carefully alternated—where in household number one, a male was selected; in the next household, a female was selected.

## Methods

The study used a mix-methods approach of qualitative and quantitative techniques. The methodology for data gathering included in-depth interviews (IDIs), focus group discussions (FGDs), and surveys using structured questionnaires. This type of study required a strong focus on individual actors rather than state actors^13^.

## Techniques of data collection

### Focus group discussions (FGDs)

These were distributed as shown in Table 1.

A set of questions covering different thematic areas were developed to guide the discussions. The questions covered healthcare services in the community, awareness of and practices for EVD, as well as assessment of the different pillars of the response interventions.

For the FGDs, 8–12 persons were selected for each session. A minimum of 2 FGDs were conducted in the selected communities. There were separate FGDs for males and females in each of the communities. Overall, a total of 8 FGDs were conducted in each province.

### In-depth Interviews (IDIs)

were conducted in each community where FGDs were carried out. Each IDI was held with community/opinion leaders in the selected communities and team leaders of response pillars. Interviews were conducted to explore people’s opinions, views, attitudes, practices, and insights regarding the outbreak and response as well as other socio-cultural factors that may influence attitudes towards the response. The FGD guide was used for the in-depth interview, focusing on the thematic areas of interest to the evaluation.

### A structured questionnaire

was used to collect quantitative data from households. The questionnaire addressed all the indicators that were used for answering the research questions. It was structured with results from the qualitative study. It was categorized into sections: socio-demographic data, perception of health problems in the community, knowledge of EVD, perceived epidemiology of Ebola in the communities, and sources of information on Ebola. Others included issues on communication and community engagement, infection prevention and control in the communities, vaccination, and surveillance as well as treatment and care. Other sets of questions covered safe and dignified burial, psychosocial issues, and logistics and security issues.

All interviews and discussions were tape-recorded with detailed notes being taken simultaneously, including verbal citations. The tape-recorded interviews were transcribed according to standard rules. Observations were also recorded. The study utilized the discussions and interviews, triangulated with the quantitative data, to arrive at its conclusions.

### Training and pilot trials

All instruments were ***translated*** into Swahili and French, the common languages spoken in the communities, and back-translated to English for clarity of meaning. In each province, ***ten research assistants*** with substantial experience in community interactive research, the use of qualitative and quantitative techniques, and cultural sensibility were recruited. These assistants then underwent six days of training—the first three in Beni and the last three in Bunia—to learn the study objectives and how to use the instruments for data collection. Training also included data entry into the Atlas.ti template (qualitative data) and EPI INFO (quantitative data). The instruments were reviewed after training for clarity, understanding, and sensitivity. Each province had a ***supervisor*** who worked with the principal investigator on data quality monitoring, safety advisory, and ethical conduct of the research including the management of informed consent procedures. The study was conducted first in Ituri, then in North Kivu. The lessons learned from Ituri were used to manage the process in North Kivu, a more security- and logistics-challenged province. A ***data analyst*** developed and pre-tested the template for data entry and analysis using the pilot test output. Given the short period of the study, data were collected using pencil and paper instead of a mobile phone. Fieldwork lasted 20 days in each province before study analysis and report writing commenced.

## Data management

### All quantitative data

were double-checked by the researcher before being entered into the computer. Data were entered into EPI Info and processed using SPSS. Descriptive statistics were used to determine the proportions of various categories of respondents and indicators and for comparison. Frequency tables and graphic illustrations were used to present the data.

### Qualitative data

obtained from FGDs and IDIs were transcribed from audio records to text. All textual data were analyzed using Atlas.ti software package. Data were analyzed according to themes corresponding to the indicators in the quantitative data and triangulated during presentation to enable complementary and analogous interpretations.

Given the continuous analytical process involved in qualitative analysis, it is important to note that the initial analysis of the key informant interviews and FGDs informed the final development of the structured questionnaire to be used in the study. This further enhanced triangulation between the two sets of data to be collected. While the quantitative results gave us statistical conclusions, the qualitative results emphasized what was actually said and provided illustrative quotes that gave context and depth to the quantitative results.

### Ethical consideration

The principle of do-no-harm was adhered to in the study. Informed study approvals were obtained at the provinces, local administration, community, and household levels, and informed consent was obtained from all individuals involved in the study. The WHO/ AFRO Ethics Review Committee provided ethical approval for the study. All researchers attended the mandatory training, which included substantial discussion of the ethical issues in research. About 50% of the research assistants were women, ensuring same-sex interviews and moderation of FGD sessions. The assistants were also trained and mandated to comply with child protection and gender sensitivity in the process of data collection and visits.

### Insecurity and disruption of the response activities

Insecurity has been a temporary obstacle to reaching some communities. Although it has often led to the cessation of activities, it has not led to the abandonment of response activities. The Ebola Treatment Centre (ETC) in Itave was destroyed. Notably, he acknowledged that he did not approve of the ETC. He claimed that the population had not been informed of the construction of the ETC saying, *“We were informed about the Ebola from people who came from Mangina. But we were surprised to see that suddenly the ETC was being built. A troop of* Maï-Maï *destroyed the ETC. Then, a security service was put in place so that there would be no more incidents.”*

Some village leaders said that the insecurity caused by armed groups in the areas of the EVD response prevented the achievement of the expected results. According to them, certain areas became inaccessible due to insecurity issues.

Insecurity derived from violence and manifested in several forms included the following:

- Attacking vaccination teams
- Attacking EVD teams
- Igniting/burning or destroying treatment facilities
- Destroying response equipment
- Attacking response vehicles and personnel
- Destroying vehicles

Insecurity led to the temporary interruption of activities in certain localities near the various response coordinating offices in Butembo, Beni, Bunia, and Mambasa. These interruptions, which delayed response activities, contributed to the failures of preventing or disrupting disease transmission—one of the main response objectives. For example, the joint teams from the epidemiological surveillance commission and the psychosocial commission were unable to work in some health facility in the Mandima health zone, where insecurity persisted.

Additionally, the work of the response teams became more challenging as contacts from the Mabalako health zone were displaced to inaccessible areas. Likewise, response activities were disrupted for several days in the Some and Mayowane health areas, which were both in the Mandima health zone. The Manguina health zone communication team reported that insecurity also affected their response work. Similarly, the health areas of Samboko and Mandibe, in the Komanda health zone, became temporarily inaccessible. During the last weeks of July, several community resistance activities occurred in the Mandima and Komanda health zones, where the SDB teams faced serious threats of bodily harm. Altercations took place between a group of young people and members of an SDB team. The team was incapable of working in the Mukuya health area in the Beni health zone. Not only was a member of the SDB team injured but the group of young people also sequestered a lifeless body that was being handled by the specialized team. In the last week of July 2019, contact monitoring could not be done as desired in several health zones.

Threats were expressed in the form of leaflets sent to the response teams in Ngogolio and Kassanga in the Beni health zone, following the abduction of a community leader and a ransom demand for his release. The threats were effective; response activities were disturbed in several health areas in the Beni sub-coordination due to insecurity demonstrated by the population. Insecurity also led to the paralysis of activities in health centers in the Vahovi health zone. Protests are often planned to object to matters unrelated to the activities of the response, as was the case in Muchanga in the city of Butembo. Planned demonstrations took place and caused response activities to cease in Beni, Butembo, Musienene, Oicha, Venda, and Lacha. While response activities were halted in each area, it was impossible to successfully conduct contract tracing, which made it difficult to detect suspected cases and carry out diagnostic activities. These shutdowns contributed to the deterioration of the epidemiological situation in the concerned areas.

Rumors are often a source of insecurity, and they also fuel attacks on the response teams and the health infrastructure. This is the case in the health area of Kalengehya. A rumor began after the death of a village girl at the Reference General Hospital (RGH) in Kyondo. Response activities were disrupted in several health areas in the Beni sub-coordination due to the insecurity caused by the population’s demonstrations. The deceased’s body was handed over to the family. The population, especially the youth, spread the rumor that certain parts of the girl’s body had been cut off. Following this rumor, the inhabitants of all the villages in that health area and its surroundings attacked the Kalengehya Health Centre, which had transferred the patient to Kyondo. The center was accused of complicity. Afterward, the population decided to make amends because the actions carried out were based on a rumor. The reason for the destruction of the health infrastructure was unfounded.

The attacks occurred in various forms. They included the destruction of facilities and equipment, such as the sorting system and the hand washing amenities in two health facilities in the city of Beni and the M’avivai health checkpoint. The Kenembahore health center in the Mutwanga health zone was attacked, and the Infection Prevention and Control (IPC) equipment was destroyed because of the rumors. These actions led the health personnel to flee this health center (Sitrep N° 356). The inhabitants of the Halangupa village went to the health center to collect the body bags to burn them. In the Katwa health zone, young people threatened to set fire to the hospitals of Muchanga and Kalengéré. In the Mambasa health zone, young people disrupted the activities of the response teams following a death in the ETC in Makanyanga.

The activities of the response teams were also interrupted in the Lwemba health area in the Mandima health zone for over two weeks in October 2019. In the first week of November 2019, the triage system of a health post in the Muyisa health area was set on fire in the Katwa health zone by unidentified people.

The insecurity even led to the displacement of part of the population in the Beni neighborhoods of Nzuma, Matembo, Ngandi, Masiani, and Boikene. Furthermore, in Beni, response activities were disrupted in the neighborhoods of Pasisi and Bundji, and hand washing amenities were destroyed in the Nzuma neighborhood. The issue of insecurity was mentioned by administrative officials, village chiefs, neighborhood chiefs, and avenue chiefs. The territorial administrator of Beni stressed that it was difficult to carry out *response* activities in the areas where they operate because of the presence of militias—such as the Mai-Mai. According to the deputy chief of the Bundji district, insecurity affected several aspects of the *response*. He said: *“Insecurity blocked the nurses in their work. Not only did it make it difficult to access patients for diagnosis, but also when the ETCs were attacked, patients could flee and infect others in the community.”*

The village chief of Kyamase spoke of massive demographic displacements, night and day, due to insecurity. According to him, this contributed to the spread of the disease. The chief of Baye Avenue in Butembo agreed. He said: *“Insecurity is among the reasons why the epidemic has not ended yet. Because of insecurity people of the* response *cannot go to the villages to raise awareness.”*

The head of Bukavu Avenue in Butembo believed that visitors who came from elsewhere because of insecurity exposed their host families to the virus as they were unaware of any illness. A community leader in Butembo spoke of the psychological effects of this insecurity. He said: *“You walk around in insecurity pretty much everywhere*.

*We become psychologically ill. When we are in a toilet, we ask ourselves if someone infected has not been here before. So, we are psychologically ill.”* He acknowledged that the epidemic created some insecurity and hoped that it would end quickly (and not last for two years).

A community leader from Boikene in Beni said that it was impossible to use some health centers or go to traditional therapists in certain places because of the safety risk.

### Insecurity and the duration of the epidemic

Community leaders played a significant role in managing insecurity. Some community leaders insisted that insecurity contributed to the deceleration of *response* activities and prolonged the epidemic. Overall, the population surveyed felt that the epidemic had lasted too long.

A large majority of the population, 65.1%, believed that the epidemic lasted too long. Many blamed it on the security situation. Slightly over a third (35.5%) of those interviewed felt that the security situation contributed to the prolongation of the epidemic.

In more than three-quarters of the cases, emigrations caused by insecurity were perceived as the main reason for the prolongation of the epidemic. This perception was also expressed in the interviews. Notably, among those who answered this question were those who mentioned political recovery. These 15 people were the ones who raised the number of respondents from 284 to 299. Insecurity was perceived as an obstacle to the activities of the response teams and as a source of psychosis for community agents. Some felt that there was insufficient information about the EVD response activities. Insecurity was also referred to as one of the greatest challenges in the response.

### Discussion

In the provinces of North Kivu and Ituri, the EVD epidemic occurred during a complex period of insecurity. From the beginning, the issue of security was handled carefully as there was no scenario in which the response could have achieved success if carried out as a military operation (according to a security officer in Goma). Many rebellious groups were active on the field, including armed groups such as the Allied Defense Forces (ADF), a Ugandan based in northern Beni. Local armed groups such as the Mai-Mai were carrying out activities that disrupted the security of the population. Thus, the security pillar of the EVD response developed a security plan for response activities in the two provinces. It was a joint plan whereby the UN Department for Safety and Security (UNDSS) collaborated with the security officers of the agencies involved in the response. Everything within this plan was completed with the support of the national security system—the defense and security forces of the DRC.

Rumors (most of which were associated with foreigner-dominant response teams) and mistrust of the intentions of the response team as well as “social resistance” (ranging from passive non-compliance to overt acts of violence) were witnessed during past EVD outbreaks as confirmed in other studies^14^. Similar scenarios also played out in Guinea; the 2014–2016 epidemic was complicated by riots, the stoning of vehicles, and even the deaths of outreach workers^14^. Some studies have enumerated factors that promoted social resistance including low levels of care in EVD treatment centers (ETCs), lack of a traditional burial for the deceased, and a distrust of foreigners “profiting” from the outbreak. Past studies during other EVD epidemics suggest that root causes of resistance or reticence relate to five domains: (1) rumors; (2) fear; (3) mistrust and lack of confidence in the authorities; (4) denial of the biomedical discourse; and (5) desire to be autonomous and avoid exogenous contamination^14,15,16^.

To implement the safety plan in North Kivu and Ituri, the national police secured the installations and workplaces of the Emergency Operations Center (EOC) in all areas of Goma, Butembo, Beni, Bunia, Mangina, and Mambasa. When response teams in the field were at risk, the security service would help them relocate and provide protection to a certain extent, but they would not use force for protection. As the head of security in Goma said, the main mission of the police is to display/demonstrate the power at their disposal. In this case, their goal was to protect the teams involved in the response and not to use force. Given that certain events expose the response personnel to challenges such as community reluctance, which can often be expressed in a violent form, several incidents have been managed in various locations.

The insecurity in these provinces led to clashes that disrupted response activities in certain localities including Beni, Mangina, and Butembo. We mentioned the role of community leaders in managing these incidents. Some incidents have led to the death of men, as reported in Bembo on April 19, 2019. The head of the Ngezi Avenue in Bunia spoke about insecurity in the context of the *response*. He said: *“It is because of the war that the treating agents cannot access all the locations. To treat the confirmed sick. If it wasn’t for this, the Ebola would still not have happened in Bunia*.”

The security pillar has taken steps to secure the work of the teams operating in the field and has addressed the presence of armed groups in the localities. This pillar focuses on strengthening the security of the personnel involved as well as training. Further, as part of the security briefing, all staff are informed about the security situation in their specific work area.

The population perceives security as a high priority in responding to the EVD outbreak, saying, for example, there is no difference between death caused by a bullet and death caused by Ebola.

The security team works with members of the risk communication and community engagement pillar. Some activities, such as immunization, are reinforced by security. Personal protective equipment (PPE) is provided to team members operating in insecure areas. Personnel are also given all types of communication equipment, including telephones and radios. Security activities are coordinated with the national party and the UN security system.

Insecurity has had a negative impact on EVD response, preventing the response from taking place in conditions that would guarantee success in a short time. The population and EVD contacts took advantage of the insecurities, fleeing to areas that were inaccessible to the response teams and, ultimately, contributing to the spread of the disease. Likewise, when communities handled Ebola victim bodies rather than accepting the practice of SDB, they contributed to the spread of the disease. Moreover, insecurity was an obstacle to the implementation of response activities, which was not optimal during the periods of disruption of the activities. This is true for all activities, but the most affected were epidemiological surveillance, risk communication and community involvement, and vaccination. Securing the response helped mitigate the impact of actions against the response teams. Accordingly, security was crucial to the response; without security, some activities would not have been impossible in communities that were often hostile to them.

### Conclusion

Insecurity, manifested in several forms, has been the primary factor in EVD response destabilization. It has prevented the work of the response teams, especially those involved in field activities and in the communities, such as the epidemiological surveillance teams, the risk communication and community engagement teams, and the teams responsible for SDB. According to most community members, insecurity has contributed to prolonging the duration of the response.

In some cases, relations between community members and the response teams were marked by altercations and even violence. Security incidents included damaging response-related equipment and facilities, such as hand washing amenities, SDB materials, and healthcare buildings. In addition, some health personnel were forced to abandon their positions due to violence from the local populations.

Protest demonstrations against the response teams were planned and carried out in several places, either by groups of young people or by the entire community. These demonstrations paralyzed response activities. Although the interruptions were often temporary, some lasted for several weeks in certain health zones. Such disruptions often led to decreased work pace of the response teams and made the tasks harder to accomplish. For example, EVD contacts moved to insecure areas that were inaccessible to the response staff, escalating the rate of disease transmission as those people who could not be checked and monitored became a more certain source of transmission. Similarly, the handling of bodies due to the refusal to accept SDB also increased the risk of transmitting EVD.

Security incidents have troubled the response. In this study, we have only described instances that took place during the period of this particular mission—from July 21 to November 2, 2019. During this period, many security-related incidents occurred across all areas of the response. However, not all efforts were impeded, as the security of the response teams enabled the implementation of activities in Butembo, Beni, Bunia, Mangina, and Mambasa among others. The Goma Community leaders contributed toward these efforts to secure the response after recognizing the threat that insecurity posed to the epidemic response. Similarly, community leaders helped establish a dialogue between members of the response and the UN Nations’ security system in the country. These collaborative security agencies involved in the response have helped overcome the non-medical obstacles to winning the fight against the EVD epidemic in the DRC.

## Figures and Tables

**Figure 1:**
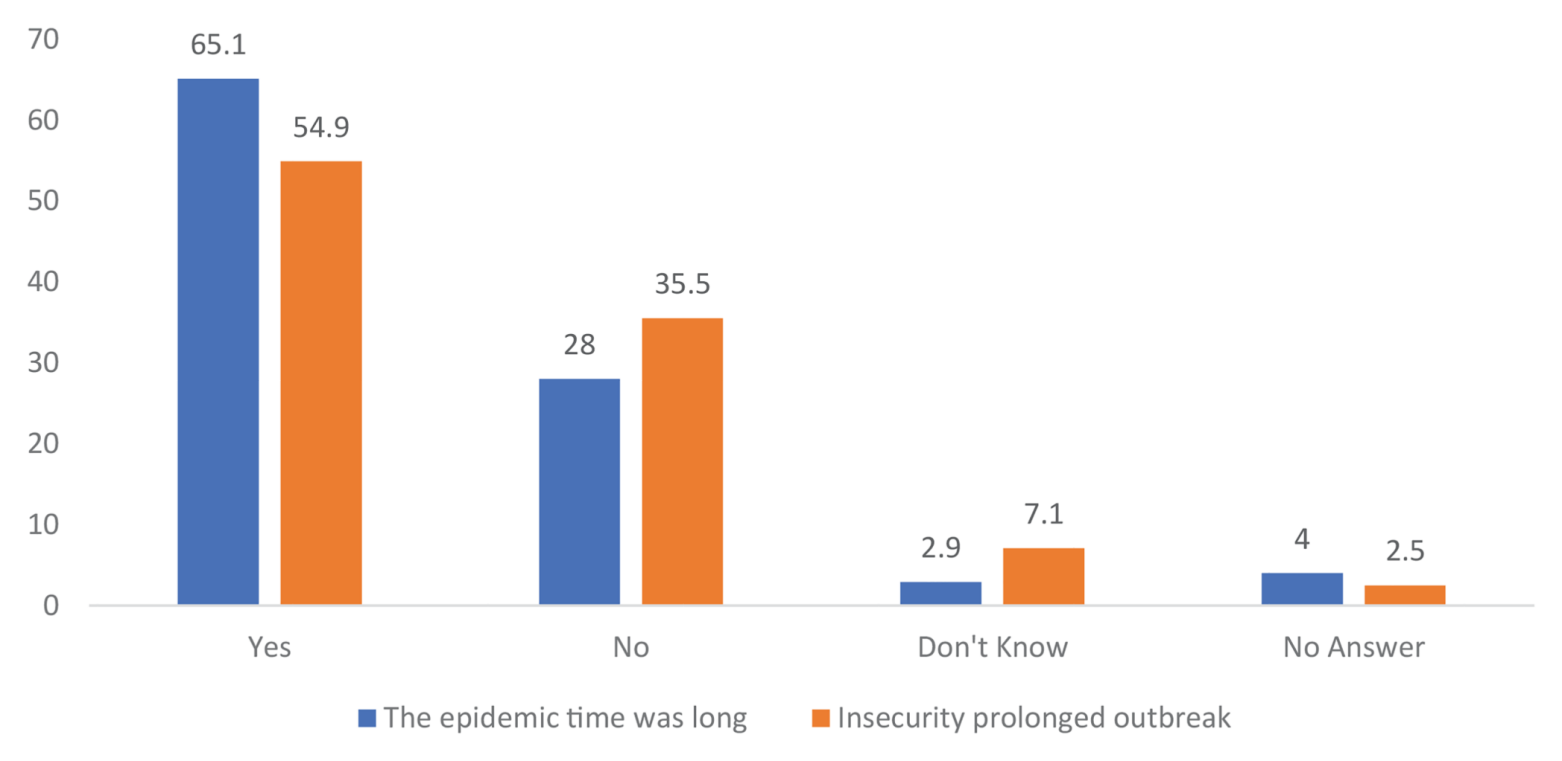
Perceptions on whether the outbreak was prolonged and whether insecurity contributed to the prolongation.

**Figure 2:**
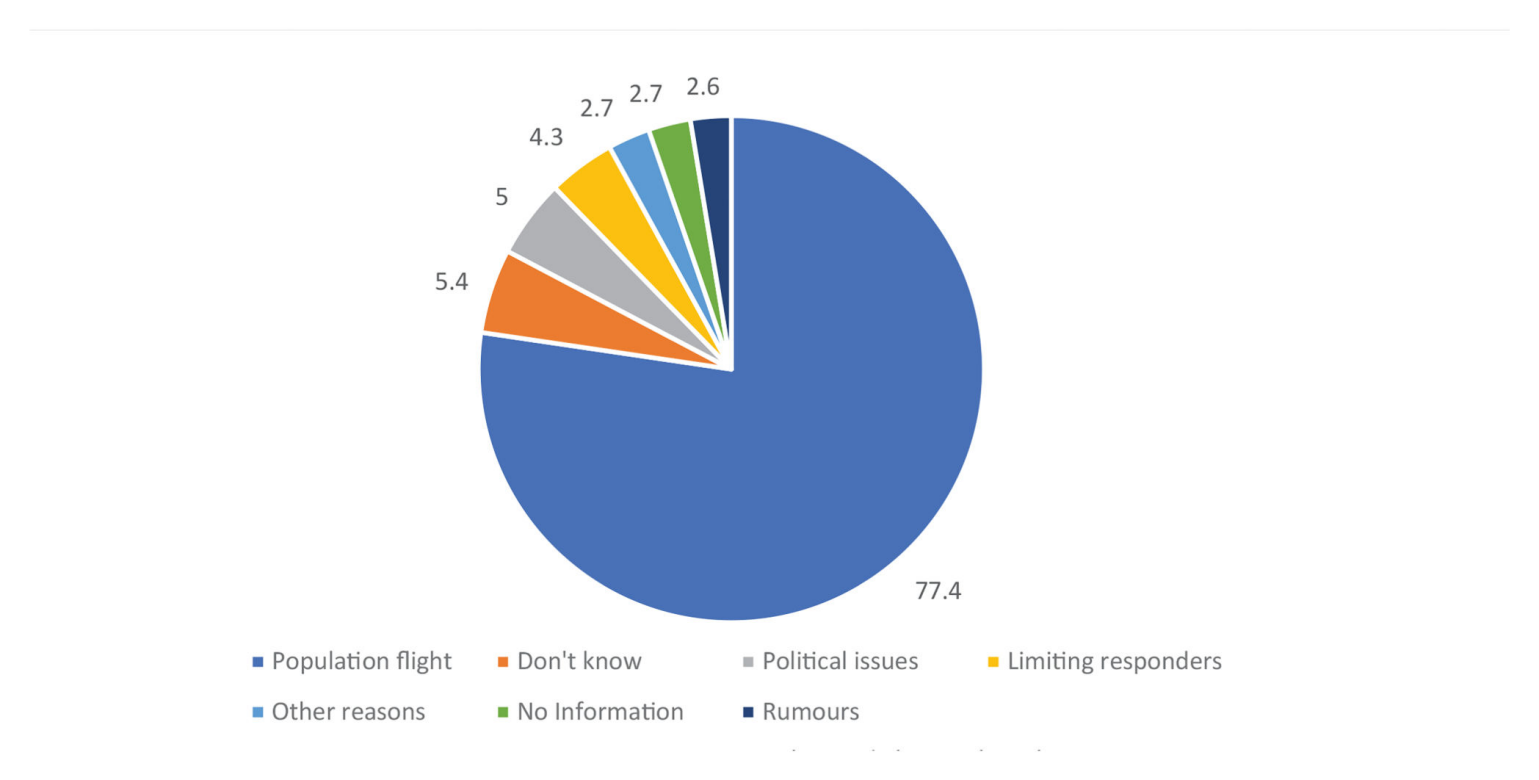
Ways insecurity prolonged the outbreak.

**Table 1:** Distribution of participants in the In-depth Interviews (IDIs) and Focus Group Discussions (FGDs) by provinces

Target	North Kivu Province				Ituri Province			
	Butembo		Beni		Mbuti		Bunia	
IDI	FGD	IDI	FGD	IDI	FGD	IDI	FGD
Pillar leads	All		All		All		All	
Pillar members	2/pillar		2/pillar		2/pillar		2/pillar	
Community leaders (trad, rel, pol, social)	≥2/ community		≥2/ Community		≥2/ community		≥2/ community	
Leader of a survivor group	≥2/ community		≥2/ Community		≥2/ community		≥2/ community	
Community adult males		≥2 groups		≥2 groups		≥2 groups		≥2 groups
Community adult females		≥2 groups		≥2 groups		≥2 groups		≥2 groups
Community male youth		≥2 groups		≥2 groups		≥2 groups		≥2 groups
Community female youth		≥2 groups		≥2 groups		≥2 groups		≥2 groups
Survivors		≥2 groups		≥2 groups		≥2 groups		≥2 groups

## Data Availability

The data that support the findings of this study are not publicly available due to their containing information that could compromise the privacy of the research participants. The data are available from the corresponding author (Joseph Okeibunor), upon reasonable request.
